# Mathematics self-efficacy and resilience link ICT use to mathematical literacy: the moderating role of mathematics anxiety

**DOI:** 10.3389/fpsyg.2026.1955686

**Published:** 2026-09-24

**Authors:** Jinhai Liu, Lulu Ren, Zhenping Wang, Shuang Gao

**Affiliations:** 1 School of Mathematics and Statistics, Henan Normal University, Xinxiang, China; 2 School of Mathematics and Statistics, Xinyang Normal University, Xinyang, China

**Keywords:** ICT use, mathematical literacy, mathematics anxiety, mathematics resilience, mathematics self-efficacy, PISA 2022

## Abstract

**Introduction:**

Digital technologies offer dynamic representations, immediate feedback, and autonomous inquiry in mathematics learning. Yet the association between ICT use and mathematical literacy may depend on students' psychological resources and emotions. Drawing on social cognitive theory, this study examined the indirect roles of mathematics self-efficacy and mathematics resilience and the moderating role of mathematics anxiety.

**Methods:**

Data were drawn from 34,406 students in PISA 2022 from Japan, Korea, Singapore, Macao, Chinese Taipei, and Hong Kong. Observed-variable path analysis tested independent and serial indirect pathways, while interaction terms and simple slope analyses assessed moderation. Analyses incorporated the final student weight and were estimated across the 10 plausible values for mathematical literacy.

**Results:**

ICT use was positively associated with mathematical literacy (β = 0.026, *p* < 0.001), mathematics self-efficacy (β = 0.311, *p* < 0.001), and mathematics resilience (β = 0.184, *p* < 0.001). Mathematics self-efficacy was positively associated with mathematics resilience (β = 0.378, *p* < 0.001) and was the strongest psychological correlate of mathematical literacy (β = 0.492, *p* < 0.001). All indirect effects were significant. The pathway through mathematics self-efficacy accounted for 81.17% of the total effect, whereas pathways involving mathematics resilience were much smaller. Mathematics anxiety weakened the association between mathematics self-efficacy and mathematical literacy (β = −0.016, *p* = 0.002) but strengthened the association between mathematics resilience and mathematical literacy (β = 0.010, *p* = 0.032).

**Discussion:**

ICT use was linked to mathematical literacy mainly through competence beliefs and adaptive psychological resources. Mathematics self-efficacy represented the central pathway, whereas mathematics resilience played a complementary role under difficulty and stress. The asymmetric moderating pattern of mathematics anxiety highlights the need for digital mathematics instruction combining technology use, task design, supportive feedback, and emotional support.

## 1 Introduction

Digital technologies are increasingly embedded in mathematics learning, expanding students' opportunities to engage in mathematical inquiry and problem solving through dynamic representations, immediate feedback, interactive manipulation, and personalized support (Liu et al., 2025). However, the availability and frequency of technology use do not necessarily translate into higher levels of learning performance. The educational value of digital technologies also depends on the purposes for which they are used, the design of learning activities, and learner characteristics (Liu and Wei, 2026; Ran et al., 2022). Therefore, examining only the direct association between information and communication technology use (ICT use) and mathematics performance is insufficient to explain individual differences in digital learning outcomes.

Previous studies have largely focused on the direct association between ICT use and mathematics achievement or mathematical literacy (Meggiolaro, 2018), but their findings have not been entirely consistent. Well-designed digital activities can support conceptual understanding, strategy adjustment, and problem solving, whereas technology use without clear learning objectives or instructional guidance may not yield positive outcomes. The educational value of ICT use may therefore depend on whether external technological support is translated into students' cognitive engagement, self-regulation, and sustained learning behavior (Courtney et al., 2022; UNESCO, 2023). This raises an important question: through which internal psychological resources is ICT use associated with mathematical literacy, and under what emotional conditions are these associations strengthened or weakened?

Social cognitive theory (SCT) provides an appropriate framework for addressing this question. The theory proposes that individual development arises from the ongoing interaction among environmental, personal, and behavioral factors (Bandura, 1986). From this perspective, ICT use constitutes an important component of the digital learning environment. The feedback, representations, and opportunities for autonomous inquiry afforded by ICT can create conditions for learning monitoring and strategy adjustment. However, whether such environmental support becomes educationally meaningful depends on how students interpret and actively use these learning opportunities (Prasse et al., 2024). The association between ICT use and mathematical literacy may therefore be explained, at least in part, by students' competence beliefs and adaptive psychological resources.

Mathematics self-efficacy (MSE) refers to students' judgments about whether they can successfully complete specific mathematics tasks. Higher MSE is generally associated with more active task engagement, more effective strategy use, greater effort, and stronger mathematics performance (Yang et al., 2024; Zakariya, 2022). In digital learning environments, visual representations, immediate feedback, and opportunities for autonomous manipulation may provide students with experiences of task mastery and success that are associated with stronger beliefs in their mathematical capabilities. Mathematics resilience (MR), by contrast, reflects students' tendency to remain engaged, continue trying, and adjust their learning strategies when facing mathematical difficulties, setbacks, or pressure (Munez et al., 2025). Whereas MSE concerns whether students believe that they can successfully complete a task, MR focuses on whether they can remain engaged and adapt after encountering difficulty.

MSE and MR may also be sequentially related. Positive judgments of one's mathematical capabilities may help students interpret difficulties as challenges that can be overcome through effort or strategic adjustment, thereby reducing avoidance and sustaining engagement. Recent research has similarly identified MSE as an important psychological factor associated with individual differences in adolescents' mathematics resilience (Wang et al., 2026). Accordingly, ICT use may first be associated with students' beliefs in their mathematical capabilities, which may subsequently be related to their persistence and strategic adaptation when facing difficulty, and ultimately to mathematical literacy. MSE and MR may therefore represent both relatively independent psychological pathways and a sequential process linking competence beliefs to adaptive responses. Integrating the two constructs within a serial framework may provide a more detailed account of how digital learning opportunities are associated with mathematical literacy through psychological resources that serve different functions.

Nevertheless, the associations between positive psychological resources and mathematical literacy may depend on students' emotional states. Mathematics anxiety (MA) refers to feelings of tension, worry, and unease experienced in mathematics learning, problem-solving, or assessment situations. Previous research has demonstrated a stable negative association between MA and mathematics performance across different age groups, measurement approaches, and mathematical tasks (Caviola et al., 2022). From a cognitive-processing perspective, anxiety-related worries and task-irrelevant thoughts consume working-memory and attentional resources, thereby reducing the efficiency of mathematical information processing and strategy execution (Finell et al., 2021). MA may therefore be associated not only directly with mathematical literacy but also with the extent to which positive psychological resources are translated into learning performance.

Because MSE and MR serve different psychological functions, MA may differentially shape their associations with mathematical literacy. MSE is a task-oriented competence belief whose role depends on whether positive self-beliefs can be translated into effective cognitive engagement and strategy execution (Gao, 2020; Rozgonjuk et al., 2020). At higher levels of anxiety, cognitive resources may be disrupted, thereby weakening the positive association between MSE and mathematical literacy. By contrast, MR is an adaptive resource oriented toward situations involving difficulty and challenge, and its role may become more salient under conditions of greater stress and pressure (Wei et al., 2026; Zhang and Cutumisu, 2024). This suggests that MA may not weaken all positive psychological resources in the same way but may instead differentially alter the strength of the associations of MSE and MR with mathematical literacy. However, few studies have examined these contrasting emotional boundary conditions within a single analytical model.

In summary, three limitations remain in the existing literature. First, the internal psychological pathways linking ICT use to mathematical literacy have not been sufficiently explained. Second, MSE and MR have generally been examined separately, and their potential sequential association has not been systematically tested. Third, it remains unclear whether MA differentially moderates the associations of competence beliefs and adaptive psychological resources with mathematical literacy. To address these issues, the present study draws on social cognitive theory and uses PISA 2022 data from students in six high-performing education systems in mathematics. A serial mediation and moderation framework was developed to examine the relationships among ICT use, MSE, MR, MA, and mathematical literacy. Specifically, the study tests the direct association between ICT use and mathematical literacy, examines the independent and serial indirect pathways involving MSE and MR, and investigates whether MA differentially moderates the associations of these two psychological resources with mathematical literacy. Given the cross-sectional nature of PISA 2022, all model pathways are interpreted as theoretically informed statistical associations rather than causal or temporal relationships. Accordingly, the following research questions were proposed:

RQ1: How is ICT use associated with students' ML?

RQ2: Do MSE and MR constitute indirect pathways linking ICT use to ML?

RQ3: Does MA moderate the associations of MSE and MR with ML?

## 2 Literature review and hypotheses

### 2.1 ICT use and mathematical literacy

ICT use is an important component of digital learning. Its educational value lies not only in providing learning resources and operational platforms, but also in extending students' mathematical cognitive activities through dynamic representations, interactive feedback, and personalized support (Meng et al., 2019). In mathematics learning, ICT can transform abstract geometric relationships, functional changes, and data structures into visual and manipulable learning objects, thereby supporting students in deepening their conceptual understanding and problem-solving abilities through observation, inquiry, and verification (Meggiolaro, 2018). The immediate feedback and adaptive practice provided by digital tools may also help students adjust their strategies according to their learning progress and make learning activities more targeted (Ghimire, 2026).

Studies based on international large-scale assessments such as PISA and TIMSS have shown that learning-oriented ICT use is generally positively associated with students' mathematics performance (Xiong et al., 2025). However, this relationship does not depend solely on the frequency of use, but may also be influenced by the purpose of use, the quality of learning activities, and learner characteristics. Although ICT use may not produce the same outcomes across all contexts, it is more likely to be associated with higher levels of ML when it supports mathematical inquiry, feedback use, and strategy adjustment. Accordingly, the following hypothesis is proposed:

*H1: ICT use is positively associated with students' ML*.

### 2.2 The mediating role of mathematics self-efficacy

Mathematics self-efficacy (MSE) refers to students' judgments of their capabilities to organize and execute the actions required to complete specific mathematical tasks (Bütüner et al., 2025). Higher levels of MSE are generally associated with greater learning engagement, more effective strategy use, more persistent task involvement, and stronger mathematics performance (Jiang et al., 2022; Yurt, 2025). In digital learning contexts, visual representations, immediate feedback, and opportunities for autonomous inquiry may provide students with experiences of task mastery and success, thereby contributing to the development of positive beliefs about their mathematical capabilities (Zhang et al., 2026).

At the same time, students with higher MSE are more likely to view ICT as a cognitive tool that supports mathematical inquiry, problem solving, and knowledge construction, rather than merely as a means of repetitive practice or superficial information retrieval (Liang et al., 2026). The learning opportunities afforded by ICT may therefore be associated with mathematical literacy through students' positive judgments of their own mathematical capabilities. Although previous studies have separately examined the relationships between ICT use and MSE and between MSE and mathematics performance, the psychological pathway through which MSE links ICT use to mathematical literacy has not yet been systematically investigated. Accordingly, the following hypothesis is proposed:

*H2: MSE mediates the association between ICT use and students' ML*.

### 2.3 The mediating role of mathematics resilience

Mathematics resilience (MR) is a domain-specific form of academic resilience that reflects students' tendency to remain engaged, continue trying, and adjust their cognitive, emotional, and learning strategies when facing difficulties, setbacks, or pressure in mathematics learning (García-Crespo et al., 2022). Students with higher levels of MR are generally better able to maintain relatively stable motivation when task difficulty increases or learning outcomes fluctuate, and they tend to demonstrate greater persistence and stronger capacity for strategic adaptation (Cheung, 2017; Martin et al., 2022).

Interactive manipulation, repeated attempts, and immediate feedback in ICT-supported environments can provide students with opportunities to identify errors, revise strategies, and cope with learning difficulties (Caponera et al., 2026). Previous research has shown that when students use ICT tools to complete mathematical tasks, they may demonstrate greater willingness to persist, stronger recognition of the value of mathematics, and more active help-seeking behavior (Chen and Wu, 2020). These characteristics may help students sustain their learning activities when encountering difficulties and transform digital learning opportunities into an ongoing process of mathematical problem solving. MR may therefore constitute a relatively independent psychological pathway linking ICT use to mathematical literacy. Accordingly, the following hypothesis is proposed:

*H3: MR mediates the association between ICT use and students' ML*.

### 2.4 The serial mediating roles of mathematics self-efficacy and mathematics resilience

Although MSE and MR are both positive psychological resources, they serve different functions. MSE reflects students' judgments of their own mathematical capabilities, whereas MR concerns their sustained engagement and adaptive regulation when facing difficulties. According to social cognitive theory, competence beliefs influence individuals' goal choices, effort, interpretations of difficulty, and coping behaviors (Bandura, 1986). MSE may therefore provide an important cognitive foundation for the development and functioning of MR, consistent with recent evidence identifying self-efficacy as an important correlate of adolescents' mathematics resilience (Wang et al., 2026). Students with higher self-efficacy are more likely to attribute mathematical difficulties to adjustable factors, such as effort, learning methods, or strategy selection, and are therefore more inclined to continue trying and revise their strategies after experiencing setbacks (Hong et al., 2026). By contrast, students with lower self-efficacy are more likely to interpret difficulties as evidence of insufficient ability, which may reduce their willingness to persist and adapt their strategies (Uslu, 2023).

In digital learning contexts, visual representations, immediate feedback, and opportunities for autonomous inquiry may be positively associated with students' sense of task mastery and MSE (Getenet et al., 2024). Stronger MSE may also be associated with students' ability to maintain engagement and adjust their strategies when they encounter errors, setbacks, or interruptions in learning, while MR may help them sustain effective learning behaviors under conditions of pressure and difficulty (Zhang et al., 2026). ICT use may therefore be associated with mathematical literacy through a theoretically specified serial indirect pathway involving competence beliefs and adaptive responses, namely, “ICT use–mathematics self-efficacy–mathematics resilience–mathematical literacy.” This ordering reflects the theoretical proposition that competence beliefs are associated with persistence and adaptive responses to difficulty; it is not intended to imply temporal precedence between MSE and MR in the present cross-sectional data. However, previous studies have generally examined MSE and MR separately, and their joint and ordered associations have received limited empirical attention. Accordingly, the following hypothesis is proposed:

*H4: ICT use is indirectly associated with students' ML through the theoretically specified serial pathway involving MSE and MR*.

### 2.5 The moderating role of mathematics anxiety

Mathematics anxiety (MA) refers to feelings of tension, worry, and unease experienced when individuals encounter mathematical tasks, solve mathematical problems, or participate in mathematics assessments. It represents a domain-specific negative emotional experience (Li et al., 2023), typically manifested in mathematics avoidance, attentional distraction, and cognitive interference (Liu et al., 2026). Previous research has shown that MA is closely associated not only with mathematics learning behaviors and performance but also with the relationships between positive psychological resources and learning outcomes (Lee, 2009; Moran-Soto and Benson, 2024).

From a cognitive resource perspective, anxiety-related worries and task-irrelevant thoughts consume working-memory and attentional resources, thereby constraining students' strategy execution and problem-solving efficiency (Jamieson et al., 2021). As a task-oriented competence belief, MSE is likely to be associated with higher mathematical literacy only when positive self-beliefs can be translated into cognitive engagement and effective strategy use. Under conditions of high anxiety, students' cognitive processing may remain disrupted even when they hold strong beliefs in their mathematical capabilities, thereby weakening the positive association between MSE and mathematical literacy (Peng and Ma, 2025). Accordingly, the following hypothesis is proposed:

*H5: MA negatively moderates the association between MSE and ML, such that the positive association becomes weaker at higher levels of MA*.

Unlike MSE, MR primarily reflects students' capacity to maintain engagement and adjust their strategies under conditions of difficulty, setbacks, and pressure. Its function may be highly context-dependent. When students experience relatively low levels of emotional pressure, the protective role of resilience may be less salient; when anxiety is high, however, persistence, strategic adjustment, and sustained engagement may become particularly important for maintaining mathematics performance (Hall et al., 2022). Thus, although higher MA is generally detrimental to mathematics learning, it may increase the relative importance of MR in relation to mathematical literacy. Accordingly, the following hypothesis is proposed:

*H6: MA positively moderates the association between MR and ML, such that the positive association becomes stronger at higher levels of MA*.

In summary, the association between ICT use and mathematical literacy may operate through students' competence beliefs and adaptive psychological resources. Drawing on social cognitive theory, the present study incorporates MSE and MR into a serial mediation pathway and conceptualizes MA as an emotional boundary condition shaping the associations between these two psychological resources and mathematical literacy. Accordingly, a theoretical model combining serial indirect and moderating pathways was developed to examine the direct association between ICT use and mathematical literacy, the independent and serial mediating roles of MSE and MR, and the differential moderating effects of MA on the two psychological resource–mathematical literacy pathways. The hypothesized model is presented in Figure 1.

**Figure 1:**
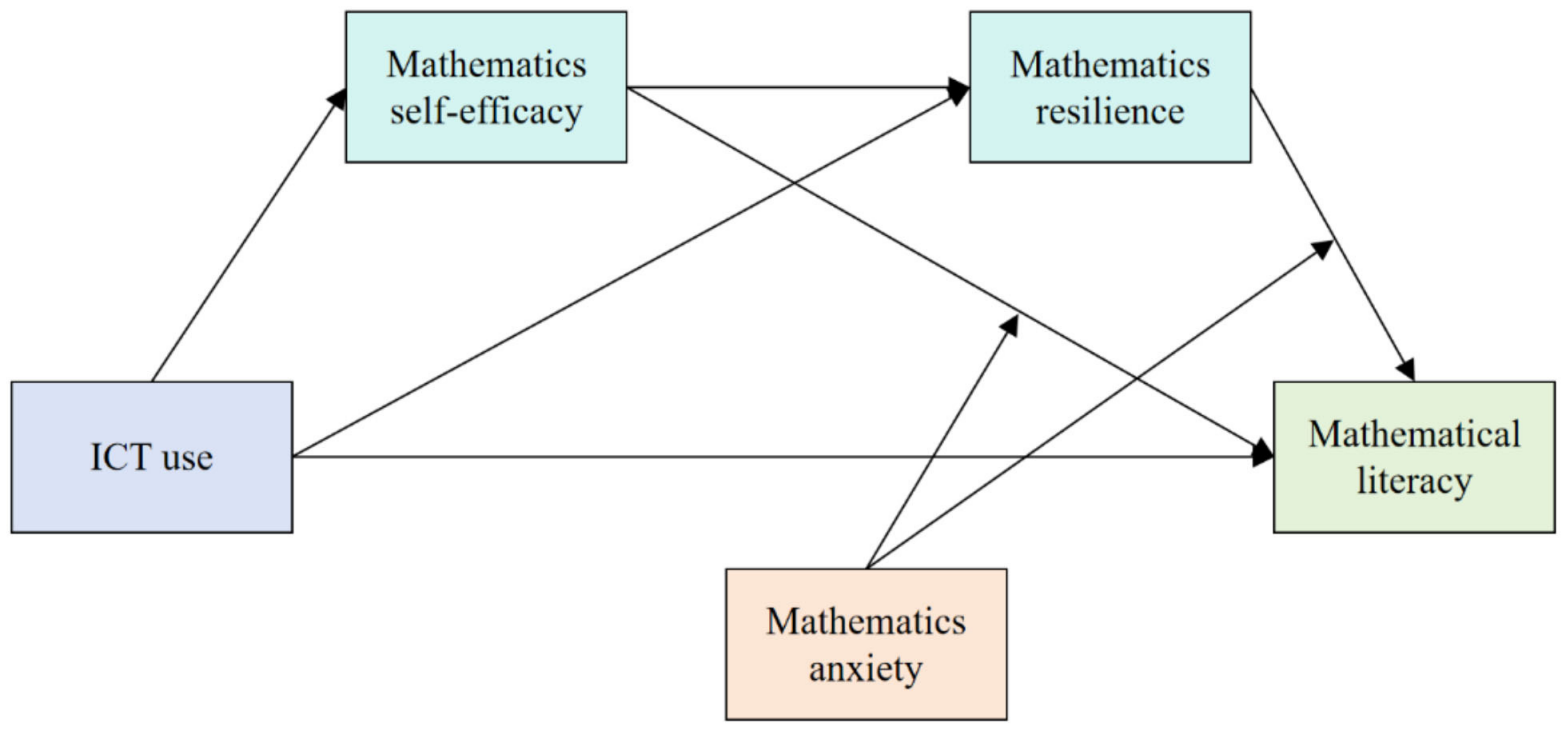
Hypothesized serial mediation model with moderated outcome paths.

## 3 Materials and methods

### 3.1 Participants and procedures

Data were obtained from the official PISA 2022 database released by the Organisation for Economic Co-operation and Development (OECD)^1^ PISA assesses the ability of 15-year-old students to apply knowledge and skills in real-world contexts, with mathematics serving as the major assessment domain in 2022. Six high-performing Asian education systems were included based on the availability of data for ICT use, mathematics self-efficacy, mathematics resilience, and mathematics anxiety, as well as sufficient sample sizes for model estimation: Japan, Korea, Singapore, Macao, Chinese Taipei, and Hong Kong. These systems were pooled to examine an overall association pattern within a set of high-performing Asian mathematics-education contexts rather than to imply cultural, institutional, or technological homogeneity. Although they share relatively high mathematics performance, they differ in educational organization, cultural expectations, and digital-learning environments. Accordingly, the pooled analysis was intended to capture aggregate associations across the selected systems rather than system-specific effects.

All eligible participants were retained in the analysis, and no cases were excluded because of missing data (0.0%). Missing values in the questionnaire variables were handled using multiple imputation. The imputation model included the focal variables and relevant background variables and generated five imputed datasets.

Within each imputed dataset, the analytical models were estimated separately using the 10 plausible values of mathematical literacy. Estimates across the plausible values were first combined, after which the results from the five imputed datasets were pooled using Rubin's rules (Rubin, 1987). The final analytical sample comprised 34,406 students. Sample characteristics by education system are presented in Table 1.

**Table 1:** Demographic characteristics of the sample by country/region.

Characteristic	Category	Japan (*N* = 5,728)	Korea (*N* = 6,371)	Singapore (*N* = 6,554)	China		
					Macao (*N* = 4,381)	Taipei (*N* = 5,786)	Hong Kong (*N* = 5,586)
Age, years	15	1,399 (24.4)	399 (6.3)	0 (0.0)	0 (0.0)	0 (0.0)	879 (15.7)
	16	4,329 (75.6)	5,972 (93.7)	6,554 (100.0)	4,381 (100.0)	5,786 (100.0)	4,707 (84.3)
Gender	Male	2,839 (49.6)	3,272 (51.4)	3,329 (50.8)	2,242 (51.2)	2,954 (51.1)	2,868 (51.3)
	Female	2,889 (50.4)	3,099 (48.6)	3,225 (49.2)	2,139 (48.8)	2,832 (48.9)	2,718 (48.7)
School level	Lower secondary	0 (0.0)	335 (5.3)	405 (6.2)	1,343 (30.7)	1,996 (34.5)	1,923 (34.4)
	Upper secondary	5,728 (100.0)	6,036 (94.7)	6,149 (93.8)	3,038 (69.3)	3,790 (65.5)	3,663 (65.6)

Values are presented as *n* (%). N denotes the total sample size for each country or region.

### 3.2 Measures

The study variables were derived from the PISA 2022 student and ICT familiarity questionnaires and the mathematics assessment. Questionnaire items were coded so that higher scores consistently represented higher levels of the corresponding constructs, with negatively worded items reverse-coded where necessary. Internal consistency was assessed using Cronbach's α, with values of 0.70 or above considered acceptable (Nunnally, 1967). The reliability coefficients reported for all study measures are interpreted according to this criterion.

#### 3.2.1 ICT use

ICT use was measured using 26 items from the PISA 2022 ICT Familiarity Questionnaire, covering four dimensions: subject-related ICT use during lessons (ICTSUBJ), ICT use in enquiry-based learning activities (ICTENQ), support or feedback via ICT (ICTFEED), and ICT use for school activities outside the classroom (ICTOUT). These dimensions reflect how frequently students use digital resources during classroom learning, enquiry-based activities, feedback and practice, and school-related activities outside the classroom.

Items were rated on a five-point frequency scale ranging from 1 (never or hardly ever) to 5 (every day or almost every day), with higher scores indicating more frequent ICT use for learning-related activities. In the present study, Cronbach's α was 0.939 across the 26 ICT items. The corresponding values for ICTSUBJ, ICTENQ, ICTFEED, and ICTOUT were 0.760, 0.926, 0.873, and 0.914, respectively, indicating acceptable to high internal consistency.

Because ICTSUBJ, ICTENQ, ICTFEED, and ICTOUT capture conceptually distinct forms and contexts of technology use, we further examined whether their aggregation into a broader ICT-use construct was psychometrically defensible. Three competing measurement models were estimated using the 26 questionnaire items: a unidimensional model, a correlated four-factor model, and a second-order model in which the four first-order ICT factors loaded on a higher-order ICT-use factor. The unidimensional model showed inadequate fit (CFI = 0.828, TLI = 0.813, RMSEA = 0.087, SRMR = 0.091), indicating that the 26 items should not be treated as a single undifferentiated dimension. In contrast, the correlated four-factor model showed good fit (CFI = 0.963, TLI = 0.959, RMSEA = 0.040, SRMR = 0.035), supporting the empirical distinction among ICTSUBJ, ICTENQ, ICTFEED, and ICTOUT. Correlations among the four factors ranged from 0.364 to 0.702. The second-order model also showed good fit (CFI = 0.966, TLI = 0.962, RMSEA = 0.039, SRMR = 0.036). Standardized loadings of the four first-order factors on the higher-order ICT-use factor were 0.486 for ICTSUBJ, 0.807 for ICTENQ, 0.803 for ICTFEED, and 0.866 for ICTOUT. Taken together, these results indicate that the four ICT dimensions are distinguishable but share sufficient common variance to support a broader higher-order ICT-use construct.

#### 3.2.2 Mathematics self-efficacy

Mathematics self-efficacy (MSE) was measured using 19 items from the PISA 2022 student questionnaire. The items covered two related dimensions: self-efficacy in formal and applied mathematics tasks, and self-efficacy in mathematical reasoning and 21st-century mathematics tasks. They assessed students' confidence in their ability to complete different types of mathematical tasks successfully. An example item was “Solve the equation 2 (*x*+3) = (*x*+3) (*x*-3)”. Items were rated on a four-point scale ranging from 1 (*not at all confident*) to 4 (*very confident*), with higher scores indicating stronger MSE. Cronbach's α was 0.974, indicating high internal consistency.

#### 3.2.3 Mathematics resilience

Mathematics resilience (MR) was operationalized using nine items from Question ST293 of the PISA 2022 student questionnaire (ST293Q01JA–ST293Q09JA). These items assess behaviors related to effort, persistence, engagement, help-seeking, and adaptive learning in mathematics and were used to construct an author-derived behavioral proxy of MR rather than an official PISA construct explicitly labeled mathematics resilience. Items were rated on a five-point frequency scale, with negatively worded items reverse-coded so that higher scores indicated stronger resilience-related behavior. Detailed item codes, conceptual mappings, and scoring procedures are provided in Appendix Table S1. Cronbach's α was 0.985.

#### 3.2.4 Mathematical literacy

Mathematical literacy was assessed using students' performance in the PISA 2022 mathematics assessment and served as the outcome variable. It reflects students' capacity to reason mathematically, apply mathematical concepts, procedures, and tools to analyze and solve problems in real-world contexts, and interpret and evaluate the resulting outcomes.

PISA 2022 provides 10 plausible values (PVs) for each student's mathematical literacy. These PVs are not deterministic individual test scores, but estimates drawn from a distribution of proficiency based on students' responses and background information. To account for measurement uncertainty, the 10 PVs were not averaged into a single score. Instead, each PV was used separately as the outcome in model estimation, and the resulting parameter estimates and variances were combined following the PISA data-analysis procedure.

#### 3.2.5 Mathematics anxiety

Mathematics anxiety (MA) was measured using six items from the PISA 2022 student questionnaire. The items assessed feelings of tension, worry, helplessness, and unease in situations involving mathematics learning, problem solving, homework, and assessment.

Items were rated on a four-point agreement scale ranging from 1 (*strongly agree*) to 4 (*strongly disagree*). All items were reverse-coded so that higher scores represented greater MA. Cronbach's α was 0.918, indicating high internal consistency.

### 3.3 Statistical analyses

Data were analyzed using SPSS 26.0 and Mplus 8.3. SPSS was used for data preprocessing, descriptive statistics, and correlation analyses, whereas Mplus was used to test the serial indirect effects, moderation effects, and simple slopes. The PISA 2022 final student weight (W_FSTUWT) was applied to adjust point estimates for unequal probabilities of selection. Replicate weights were not incorporated into variance estimation in the present analysis. Mathematical literacy was represented by 10 PVs. For each of the five multiply imputed datasets, the same analytical model was estimated separately using each of the 10 PVs, yielding 50 model estimations in total. Within each imputed dataset, parameter estimates were first combined across the 10 PVs. The total variance incorporated both the average within-PV variance and the between-PV variance, thereby accounting for uncertainty associated with the plausible-value representation of mathematical literacy. The resulting estimates from the five multiply imputed datasets were then pooled using Rubin's rules, which incorporated the additional between-imputation uncertainty associated with missing questionnaire data. Final standard errors were derived from the corresponding pooled variance estimates, and 95% confidence intervals were calculated from the pooled estimates and standard errors. All statistical tests were two-tailed, and p < 0.05 was considered statistically significant (Hair et al., 2009).

The analyses proceeded in three steps. First, weighted means, standard deviations, and correlations were calculated. Second, a serial mediation model was estimated to examine the direct association between ICT use and mathematical literacy, together with three specific indirect pathways: ICT use → mathematics self-efficacy → mathematical literacy; ICT use → mathematics resilience → mathematical literacy; and ICT use → mathematics self-efficacy → mathematics resilience → mathematical literacy. Standardized coefficients were reported for individual paths, whereas total, direct, and indirect effects were reported as unstandardized estimates. Bias-corrected bootstrap confidence intervals were generated using 5,000 individual-observation-level resamples. These bootstrap analyses were conducted within models incorporating the final student weight (W_FSTUWT), but the resampling procedure was not combined with the PISA replicate weights and therefore did not reproduce the full complex sampling structure of PISA. Accordingly, the resulting bootstrap confidence intervals should be interpreted as model-based rather than fully design-based PISA confidence intervals.

Finally, interaction terms were created for mathematics self-efficacy × mathematics anxiety and mathematics resilience × mathematics anxiety to test whether mathematics anxiety moderated the associations of the two psychological resources with mathematical literacy. Significant interactions were further examined through simple slope analyses at low, mean, and high levels of mathematics anxiety. The 95% confidence intervals for the conditional effects were estimated using the same 5,000-resample bootstrap procedure and are subject to the same design-based limitation described above. Given the cross-sectional nature of PISA 2022, all findings were interpreted as statistical associations rather than causal or temporal relationships.

## 4 Results

### 4.1 Descriptive statistics and correlation analysis

Table 2 presents the weighted descriptive statistics and correlations among the study variables for the pooled sample. ICT use, MSE, and MR were all significantly and positively correlated with ML. MSE showed the strongest correlation with ML (*r* = 0.507, *p* < 0.01), followed by MR (*r* = 0.237, *p* < 0.01) and ICT use (*r* = 0.184, *p* < 0.01).

**Table 2:** Descriptive statistics and correlations among the study variables.

Variable	M	SD	1	2	3	4	5
ICT use	−0.15	0.82	1				
MSE	−0.20	1.10	0.311^**^	1			
MR	−0.16	1.02	0.302^**^	0.435^**^	1		
ML	546.36	99.72	0.184^**^	0.507^**^	0.237^**^	1	
MA	0.20	1.15	−0.032^**^	−0.404^**^	−0.232^**^	−0.232^**^	1

M, mean; SD, standard deviation. ^**^p < 0.01.

Among the psychological variables, ICT use was positively correlated with both MSE (*r* = 0.311, *p* < 0.01) and MR (*r* = 0.302, *p* < 0.01), while MSE and MR were moderately positively correlated (*r* = 0.435, *p* < 0.01). MA was negatively correlated with MSE (*r* = −0.404, *p* < 0.01), MR (*r* = −0.232, *p* < 0.01), and ML (*r* = −0.232, *p* < 0.01), and showed a weak negative correlation with ICT use (*r* = −0.032, *p* < 0.01). Overall, the directions of the correlations were consistent with the theoretical expectations, and no excessively high bivariate correlations were observed, providing preliminary support for the subsequent analyses of the serial indirect pathways and moderation effects.

### 4.2 Mediation effect testing

The serial mediation model was examined using regression-based path analysis, with the results presented in Figure 2 and Tables 3, 4 (Figure 1 presents the full hypothesized model, including both the serial indirect and moderation pathways, whereas Figure 2 reports the estimated standardized coefficients for the serial mediation component only. The moderation effects are presented separately in Figures 3, 4 for clarity.). ICT use was positively associated with mathematics self-efficacy (MSE; β = 0.311, *p* < 0.001), explaining 9.7% of its variance. Both ICT use (β = 0.184, *p* < 0.001) and MSE (β = 0.378, *p* < 0.001) were positively associated with mathematics resilience (MR), jointly explaining 22.0% of its variance. In the ML model, ICT use (β = 0.026, *p* < 0.001), MSE (β = 0.492, *p* < 0.001), and MR (β = 0.015, *p* = 0.027) were all positively associated with ML. The model explained 25.8% of the variance in ML. MSE showed the largest standardized association with ML, whereas the direct associations of ICT use and MR with ML were comparatively weak. Thus, H1 was supported.

**Figure 2:**
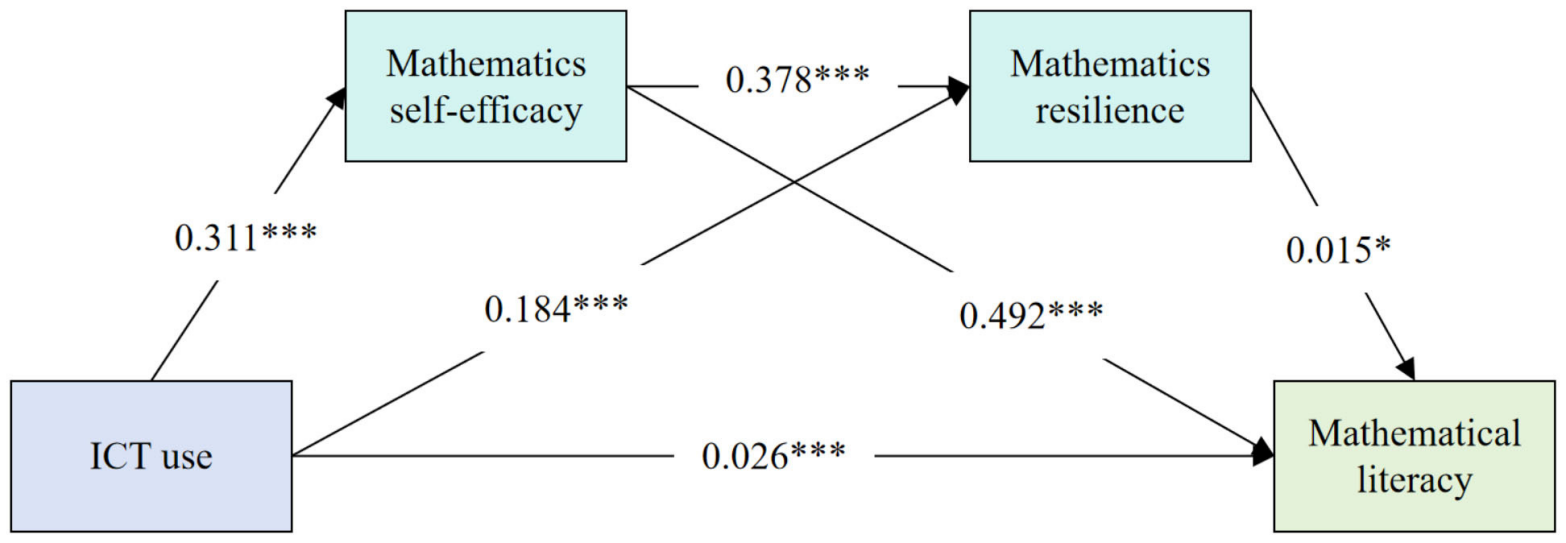
Estimated standardized path coefficients for the serial mediation component of the hypothesized model.

**Table 3:** Regression results for the serial mediation model.

Outcome	Predictor	R	R^2^	F	β	t	*p*
MSE	ICTU	0.311	0.097	3,677.533^***^	0.311	60.643^***^	< 0.001
MR	ICTU	0.469	0.220	4,847.982^***^	0.184	36.798^***^	< 0.001
	MSE				0.378	75.377^***^	< 0.001
ML	ICTU	0.508	0.258	3,980.317^***^	0.026	5.272^***^	< 0.001
	MSE				0.492	93.232^***^	< 0.001
	MR				0.015	2.919^*^	0.027

ICTU, ICT use; MSE, mathematics self-efficacy; MR, mathematics resilience; ML, mathematical literacy. ^*^p < 0.05. ^***^p < 0.001.

**Table 4:** Total, direct, and indirect effects of the serial mediation model.

Path	Effect estimate	*SE*	95% CI	Proportion of total effect
Total effect	47.472	1.616	[44.389, 50.690]	—
Direct effect	7.966	1.501	[5.062, 10.986]	16.78%
Total indirect effect	39.506	1.022	[37.494, 41.533]	83.22%
ICTU → MSE → ML	38.532	0.980	[36.656, 40.533]	81.17%
ICTU → MR → ML	0.614	0.280	[0.050, 1.148]	1.29%
ICTU → MSE → MR → ML	0.360	0.163	[0.028, 0.671]	0.76%

ICTU, ICT use; MSE, mathematics self-efficacy; MR, mathematics resilience; ML, mathematical literacy; SE, standard error; CI, confidence interval. The proportion of each effect was calculated relative to the total effect.

**Figure 3:**
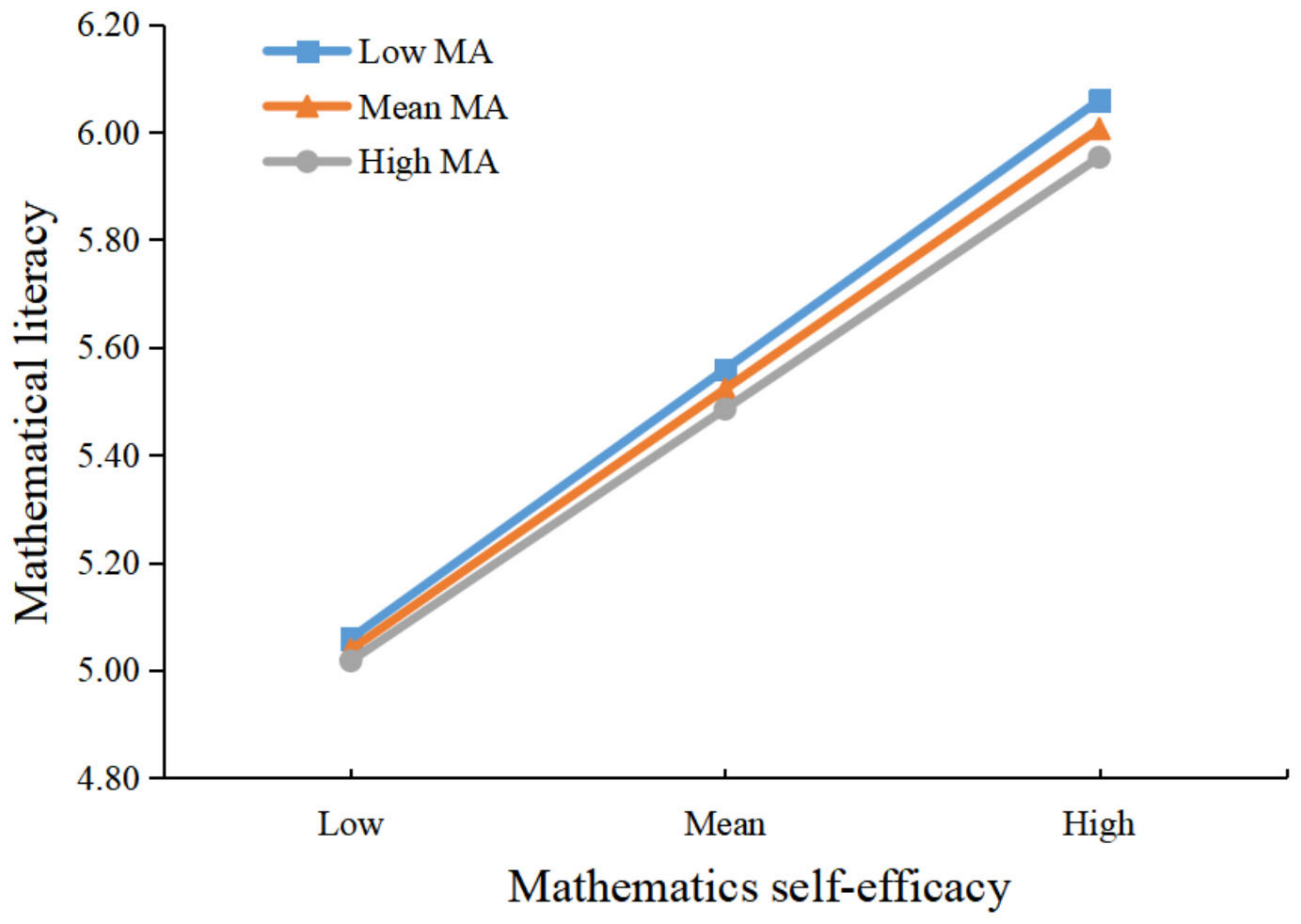
Interaction between mathematics self-efficacy and mathematics anxiety in predicting mathematical literacy. Predicted mathematical literacy scores are presented on a scale divided by 100 for graphical clarity; the analyses were conducted using the original PISA score metric.

**Figure 4:**
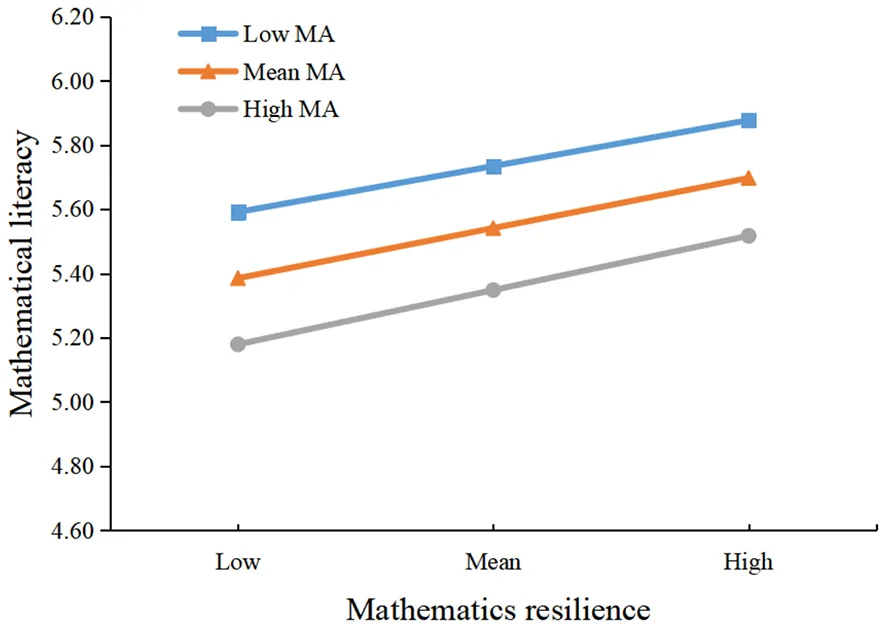
Interaction between mathematics resilience and mathematics anxiety in predicting mathematical literacy. Predicted mathematical literacy scores are presented on a scale divided by 100 for graphical clarity; the analyses were conducted using the original PISA score metric.

The indirect-effect results are presented in Table 4. The unstandardized total effect of ICT use on ML was 47.472, with a 95% confidence interval of [44.389, 50.690]. After MSE and MR were included, the direct effect remained statistically significant (effect = 7.966, 95% CI [5.062, 10.986]) and accounted for 16.78% of the total effect. The total indirect effect was 39.506, with a 95% confidence interval of [37.494, 41.533], representing 83.22% of the total effect. This indicates that, within the proposed model, most of the association between ICT use and ML operated through the indirect pathways involving MSE and MR.

More specifically, the indirect effect through MSE was the largest (effect = 38.532, 95% CI [36.656, 40.533]). Within the specified cross-sectional model, the indirect association involving MSE represented 81.17% of the model-estimated total association between ICT use and mathematical literacy, supporting H2. The indirect effect through MR was 0.614 (95% CI [0.050, 1.148]), accounting for 1.29% of the total effect and supporting H3. In addition, the serial indirect effect through ICT use → MSE → MR → ML was 0.360 (95% CI [0.028, 0.671]), accounting for 0.76% of the total effect and supporting H4. The confidence intervals for all three specific indirect effects excluded zero.

Overall, MSE represented the primary psychological pathway linking ICT use to ML. By contrast, although the indirect effect through MR and the serial indirect effect were statistically significant, both were small in magnitude. Accordingly, the MR-related findings should be interpreted in light of both their statistical significance and their limited practical effect sizes.

### 4.3 Moderation effect testing

The moderation results are presented in Table 5. In the MSE moderation model, the interaction between MSE and MA was negatively associated with ML (β = −0.016, *t* = −3.046, *p* = 0.002), indicating that the positive association between MSE and ML weakened as MA increased. This result was consistent with the negative moderation proposed in H5; therefore, H5 was supported.

**Table 5:** Regression results for the moderation models.

Outcome	Predictor	*R*	*R* ^2^	*F*	β	*t*	*p*
ML	MSE	0.508	0.258	5,968.659^***^	0.484	96.037^***^	< 0.001
	MA				−0.037	−6.723^***^	< 0.001
	MSE × MA				−0.016	−3.046^**^	0.002
ML	MR	0.299	0.089	1,687.643^***^	0.156	25.749^***^	< 0.001
	MA				−0.193	−34.403^***^	< 0.001
	MR × MA				0.010	2.147^*^	0.032

MSE, mathematics self-efficacy; MR, mathematics resilience; MA, mathematics anxiety; ML, mathematical literacy. ^*^p < 0.05. ^**^p < 0.01. ^***^p < 0.001.

In the MR moderation model, the interaction between MR and MA was positively associated with ML (β = 0.010, *t* = 2.147, *p* = 0.032). This result was consistent with the positive moderation proposed in H6; therefore, H6 was supported. Although both interaction terms reached statistical significance, their standardized coefficients were small (β = −0.016 and β = 0.010), indicating limited moderation effects. Simple slope analyses were therefore conducted to further examine these interactions.

To further interpret the interaction effects, simple slope analyses were conducted at low (one standard deviation below the mean), mean, and high (one standard deviation above the mean) levels of MA. The results are presented in Table 6 and Figures 3, 4. The conditional association between MSE and ML was statistically significant at all three levels of MA, with standardized slopes of 0.499, 0.484, and 0.468, respectively (all *p* < 0.001). Thus, the positive association between MSE and ML gradually weakened as MA increased.

**Table 6:** Conditional effects at different levels of mathematics anxiety.

Outcome	Predictor	Level of MA	β	t	Bootstrap 95% CI	
					Lower	Upper
ML	MSE	low (M – 1 SD)	0.499	43.128^***^	0.478	0.502
		mean (M)	0.484	44.892^***^	0.467	0.491
		high (M + 1 SD)	0.468	40.585^***^	0.451	0.480
ML	MR	low (M – 1 SD)	0.143	17.182^***^	0.127	0.159
		mean (M)	0.156	22.483^***^	0.141	0.165
		high (M + 1 SD)	0.170	19.726^***^	0.149	0.179

^*^p < 0.001.

The conditional association between MR and ML was also statistically significant at low, mean, and high levels of MA, with standardized slopes of 0.143, 0.156, and 0.170, respectively (all *p* < 0.001). In contrast to the pattern observed for MSE, the positive association between MR and ML strengthened as MA increased.

Overall, MA did not change the directions of the associations of MSE and MR with ML but differentially altered their strength. Higher MA weakened the positive association between MSE and ML while strengthening the positive association between MR and ML. However, both moderation effects were small and should therefore be interpreted cautiously in light of their practical magnitude.

## 5 Discussion

### 5.1 ICT use and mathematical literacy

The results showed that ICT use remained positively associated with mathematical literacy (ML) after accounting for mathematics self-efficacy (MSE) and mathematics resilience (MR), supporting H1. This finding is consistent with previous PISA-based research showing that learning-oriented ICT use is generally associated with better mathematics performance (Sanfo, 2023). Digital tools can provide dynamic representations, immediate feedback, and interactive manipulation, helping students move among symbolic, graphical, and real-world representations and engage in mathematical problem solving through observation, verification, and strategy adjustment (Weigand et al., 2024). The positive association between ICT use and ML may therefore reflect its support for mathematical representation, feedback use, and cognitive engagement (Cai et al., 2023; Ran et al., 2022; Weigand et al., 2024).

However, the direct association was weak, suggesting that the frequency of ICT use alone does not adequately explain differences in ML. This finding is inconsistent with a simple linear assumption that greater technology use necessarily corresponds to better learning outcomes (Gorjón and Osés, 2023). The educational relevance of ICT may depend on the purpose of use, task design, instructional guidance, and students' self-regulation. Technology use without clear learning goals may also distract attention or increase extraneous cognitive load (Zhu and Li, 2022). Accordingly, the key issue in digital mathematics learning may not be simply increasing ICT use, but improving the instructional quality of technology-supported activities. ICT is more likely to be associated with higher ML when embedded in purposeful and appropriately challenging tasks that provide effective feedback and encourage reasoning, explanation, and strategy adjustment (Radović, 2024).

The timing of PISA 2022 also warrants consideration when interpreting this association. The assessment followed substantial pandemic-related disruption to in-person schooling, during which digital technologies became especially important for maintaining learning continuity. Students differed in their access to digital resources, familiarity with online learning, and preparedness to use ICT for school-related activities (OECD, 2023). Students with greater access to, and greater confidence in using, digital technologies may therefore have been better positioned to sustain their mathematics learning during this period. Accordingly, the positive association between ICT use and ML observed in PISA 2022 may partly reflect this historically specific educational context and the expanded role of technology-supported learning during and immediately following pandemic-related disruptions.

At the same time, the cross-sectional nature of the data does not establish the direction of the observed association. Although the proposed model was theoretically specified from ICT use to ML, the reverse interpretation is also plausible. Students with higher mathematical literacy may be better able to use digital tools strategically for mathematical inquiry, problem solving, and feedback seeking, or may be more inclined to engage in ICT-supported learning activities. The observed relationship may therefore reflect reciprocal or selection processes rather than a unidirectional influence of ICT use on mathematical literacy. Longitudinal research is needed to clarify the temporal ordering and potential reciprocal relationships between ICT use and mathematical literacy.

### 5.2 Serial mediating roles of mathematics self-efficacy and mathematics resilience

The results showed significant indirect associations involving MSE and MR, both independently and through a serial pathway linking ICT use with ML. This finding is consistent with previous research indicating that MSE is related not only to mathematics performance but also to students' task choices, effort, and persistence (Liao and Zhang, 2023; Street et al., 2024). In digital learning contexts, students' ability to use technology-supported feedback, representations, and learning options effectively depends partly on their judgments of their own mathematical competence (Cai et al., 2023; Smit et al., 2023). When tasks are appropriately challenging and feedback is explanatory and supports strategy adjustment, digital learning is more likely to provide successful learning experiences associated with stronger MSE (Kuklick et al., 2023).

MSE represented a substantially stronger indirect pathway than MR, suggesting that competence beliefs may be particularly important in understanding the association between digital learning experiences and mathematical outcomes. Previous studies similarly indicate that self-efficacy is closely related to sustained engagement and deeper learning in technology-enhanced settings (Han and Geng, 2023; Tang et al., 2021). At the same time, the present cross-sectional findings allow for alternative interpretations. Mathematics self-efficacy is embedded within a broader constellation of competence beliefs, motivation, and mathematics-related emotions that are themselves closely associated with mathematics achievement (Lee, 2009; Zou, 2025). Students with stronger general academic confidence, or confidence in mathematics and STEM-related abilities more broadly, may be more willing to engage with digital learning tools while also being more likely to perform well in mathematics. Thus, the strong indirect association involving MSE may partly reflect broader confidence-related characteristics that were not explicitly represented in the present model.

By contrast, MR may become particularly relevant when students encounter errors, setbacks, or stalled progress, helping them maintain engagement and adjust their strategies. Its relatively small indirect effect therefore does not imply limited value, but may reflect its more context-dependent protective function (Mills, 2021). The serial indirect association was consistent with the theoretically specified ordering from competence beliefs to adaptive learning responses. ICT use was positively associated with MSE, MSE was positively associated with MR, and both psychological resources were associated with mathematical literacy. However, because these variables were measured contemporaneously, this statistical ordering should not be interpreted as evidence that MSE temporally precedes MR, that ICT use temporally precedes either psychological resource, or that these variables causally transmit the association between ICT use and ML. Rather, the findings indicate that ICT use, competence beliefs, adaptive responses, and mathematical literacy are systematically related within the proposed theoretical framework. Thus, the educational relevance of digital technology may depend not only on access to resources but also on how digital learning is associated with students' competence judgments and adaptive responses to difficulty.

### 5.3 Differential moderating role of mathematics anxiety

Mathematics anxiety (MA) was not only negatively associated with mathematical literacy (ML) but also differentially moderated the associations of mathematics self-efficacy (MSE) and mathematics resilience (MR) with ML. This finding is consistent with previous evidence that the negative association between MA and mathematics performance occurs across age groups, tasks, and educational contexts (Barroso et al., 2021; Liang et al., 2025). The present study further indicates that MA constitutes an important emotional boundary condition shaping how positive psychological resources relate to ML. More broadly, previous research has shown that mathematics achievement is embedded within a wider affective-motivational system in which positive emotions, self-efficacy, self-regulation, and mathematics anxiety are closely interconnected (Villavicencio and Bernardo, 2016).

Specifically, the positive association between MSE and ML weakened as MA increased. This interpretation is consistent with evidence that domain-specific self-efficacy and anxiety are closely intertwined, with perceived self-efficacy playing an important role in students' emotional and motivational responses to quantitatively demanding learning tasks (Kaufmann et al., 2022). Anxiety-related worries and task-irrelevant thoughts can consume working-memory and attentional-control resources, thereby reducing the efficiency of mathematical information processing and strategy execution (Cuder et al., 2023; Shi et al., 2022). Consequently, even students with positive beliefs about their mathematical competence may struggle to translate those beliefs into mathematics performance when experiencing high anxiety. This suggests that the role of MSE is constrained by students' emotional states: lower anxiety may facilitate the translation of competence beliefs into effective engagement, whereas higher anxiety may disrupt this process.

By contrast, the positive association between MR and ML was stronger at higher levels of MA. This pattern is consistent with the view that positive psychological resources become particularly protective under stressful conditions (Putwain et al., 2020; Putwain and Wood, 2023). When anxiety is low, emotional threat is limited and the role of resilience may be less salient. Under high anxiety, however, students may be more prone to avoidance, disengagement, and attentional distraction, making sustained effort and strategic adjustment increasingly important (Doz et al., 2024). Nevertheless, MR may only partially buffer anxiety-related disruptions and cannot eliminate the cognitive burden associated with MA.

Overall, the asymmetric moderating patterns further highlight the functional distinction between MSE and MR. MSE represents a task-oriented competence belief whose value depends on whether confidence can be translated into effective cognitive processing. MR, in contrast, is an adaptive resource oriented toward managing difficulty and may become more important under conditions of heightened pressure. Thus, MA does not uniformly weaken all positive psychological resources; rather, its moderating role varies according to the specific function of each resource.

The magnitude of these associations should also be considered alongside their statistical significance. Given the large sample size, several statistically significant coefficients were small, particularly the direct associations of ICT use and MR with mathematical literacy and the two interaction effects. These results therefore indicate statistically detectable associations, but their practical educational importance should be interpreted cautiously.

### 5.4 Theoretical and practical implications

The findings offer several tentative theoretical implications. First, the observed pattern is consistent with applying a social cognitive perspective to digital mathematics learning by considering ICT use, competence beliefs, adaptive resources, and domain-specific emotions within a common framework. Second, the indirect associations involving MSE and MR suggest that students' psychological resources may be relevant to understanding how ICT use is associated with mathematical literacy. Third, the contrasting interaction patterns involving MA suggest that domain-specific emotions may condition these associations, although the small effect sizes warrant cautious interpretation.

From a practical perspective, the findings suggest that attention to the quality of technology-supported learning activities may be as important as access to or frequency of ICT use. Purposeful mathematical tasks, explanatory feedback, and strategy support may support students' competence beliefs and adaptive responses to difficulty. These implications should be viewed as directions for educational practice and future intervention research rather than as effects established by the present cross-sectional data.

## 6 Limitations and future directions

Despite using a large PISA 2022 sample and incorporating the final student weight and plausible values for mathematical literacy, this study has several limitations. First, the cross-sectional design precludes causal or temporal inferences. Although the proposed ordering of variables was theoretically informed, alternative explanations remain plausible. For example, higher mathematical literacy may facilitate more effective ICT use, while broader academic or STEM-related confidence may underlie both ICT engagement and mathematics outcomes. The identified indirect pathways should therefore be interpreted as theory-informed associations rather than causal mediation. Future longitudinal or cross-lagged studies incorporating broader confidence measures are needed to clarify the temporal ordering of these relationships.

Second, the timing of PISA 2022 should be considered when interpreting the observed association between ICT use and mathematical literacy. The assessment followed substantial pandemic-related disruptions to in-person schooling, during which students relied more heavily on digital technologies and online learning. Students with greater access to digital resources and greater familiarity with ICT-supported learning may have been better positioned to sustain their mathematics learning during this period. Thus, the observed ICT–mathematical literacy association may partly reflect this historically specific educational context. Future studies using later cohorts are needed to examine whether this pattern remains stable under more typical schooling conditions.

Third, ICT use and the psychological variables were based on student self-reports, which may be subject to response bias and shared-method variance. Although these measures capture overall use, they cannot fully distinguish purposes, activity quality, teacher guidance, or device types. Future studies could combine questionnaire data with platform logs, task records, screen-based behavioral data, and classroom observations (Ober et al., 2021) to differentiate the frequency and quality of ICT use and compare the effects of specific digital activities.

Fourth, the study focused on six high-performing education systems. While this provides insight into digital learning in high-achievement contexts, the findings may not generalize to educational systems with substantially different cultural, socioeconomic, or technological conditions. Future research should include a broader range of education systems and assess cross-cultural stability through measurement invariance testing and multigroup analysis.

Finally, several statistically significant associations were small in magnitude and should therefore be interpreted with attention to their practical significance. In addition, although the final student sampling weight was incorporated, the present analyses did not use the full set of PISA replicate weights for variance estimation. Consequently, the reported standard errors, confidence intervals, and significance tests may not fully reflect uncertainty arising from the complex PISA sampling design. This limitation is particularly relevant for small coefficients or associations with *p* values close to conventional significance thresholds, which should therefore be interpreted with additional caution. The model also did not systematically include background factors such as socioeconomic status, gender, school type, and school resources. Some observed associations may therefore be influenced by confounding variables. Future studies should incorporate theoretically relevant covariates and evaluate robustness using subgroup analyses, alternative models, and sensitivity analyses.

## 7 Conclusion

Using student data from six high-performing education systems participating in PISA 2022, this study examined the serial indirect pathways linking ICT use to mathematical literacy and the moderating role of mathematics anxiety. ICT use was positively associated with mathematical literacy, although the direct association was relatively weak. Most of the association operated indirectly through MSE and MR. MSE represented the primary psychological pathway and was further linked to mathematical literacy through MR. MA served as a differential emotional boundary condition: higher MA weakened the positive association between MSE and mathematical literacy but strengthened the positive association between MR and mathematical literacy. These findings suggest that the educational value of digital technology may depend not only on its use, but also on students' competence beliefs, adaptive learning responses, and emotional states. Digital mathematics instruction should therefore integrate technology use, task design, and psychological support, shifting from an emphasis on technology use alone toward the development of students' sustainable capacity for mathematics learning.

## Data Availability

Publicly available datasets were analyzed in this study. The PISA 2022 data are available from the OECD PISA 2022 database at https://www.oecd.org/en/data/datasets/pisa-2022-database.html.

## Footnotes

1 https://www.oecd.org/en/data/datasets/pisa-2022-database.html.

## Appendix

**Table A1:** PISA 2022 items used to operationalize mathematics resilience.

Item code	Item content (brief description)	Resilience-related interpretation	Scoring	Reverse-coded
ST293Q01JA	Active participation in mathematics class discussions	Sustained/active engagement	1–5	No
ST293Q02JA	Paying attention during mathematics instruction	Sustained engagement	1–5	No
ST293Q03JA	Effort devoted to mathematics assignments	Effort and persistence	1–5	No
ST293Q04JA	Giving up when mathematics material is not understood	Persistence under difficulty	1–5	Yes
ST293Q05JA	Making time to learn mathematics material	Sustained effort	1–5	No
ST293Q06JA	Asking questions when mathematics material is not understood	Adaptive help-seeking	1–5	No
ST293Q07JA	Losing interest during mathematics lessons	Sustained engagement	1–5	Yes
ST293Q08JA	Connecting new mathematics material with prior learning	Adaptive learning strategy	1–5	No
ST293Q09JA	Starting mathematics assignments promptly	Proactive engagement	1–5	No
